# Identifying risk factors for perinatal death at Tororo District Hospital, Uganda: a case-control study

**DOI:** 10.1186/s12884-020-2727-3

**Published:** 2020-01-20

**Authors:** Martha A. Tesfalul, Paul Natureeba, Nathan Day, Ochar Thomas, Stephanie L. Gaw

**Affiliations:** 1 Division of Maternal-Fetal Medicine, Department of Obstetrics, Gynecology and Reproductive Sciences, University of California, San Francisco, 550 16th St, Box 0132, San Francisco, CA 94143 USA; 2 Infectious Diseases Research Collaboration, Plot 2C, Nakasero Hill, P.O. Box 7475, Kampala, Uganda; 3 Current address: Makerere University –Johns Hopkins University Collaboration, Upper Mulago Hill Road, P.O. Box 23491, Kampala, Uganda; 4 Tororo District Hospital, Station Road, P.O. Box 2, Tororo, Uganda; 5 Department of Obstetrics, Gynecology and Reproductive Sciences, University of California, San Francisco, 513 Parnassus Ave, Box 0556, 16HSE, San Francisco, CA 94143 USA

**Keywords:** Stillbirth, Neonatal death, Obstetrics, Global health, Africa

## Abstract

**Background:**

Sub-Saharan Africa faces a disproportionate burden of perinatal deaths globally. However, data to inform targeted interventions on an institutional level is lacking, especially in rural settings. The objective of this study is to identify risk factors for perinatal death at a resource-limited hospital in Uganda.

**Methods:**

This is a retrospective case-control study at a district hospital in eastern Uganda using birth registry data. Cases were admissions with stillbirths at or beyond 24 weeks or neonatal deaths within 28 days of birth. Controls were admissions that resulted in deliveries immediately preceding and following each case. We compared demographic and obstetric factors between cases and controls to identify risk factors for perinatal death. Subgroup analysis of type of perinatal death was also performed. Chi square, Fisher’s exact, t-test, and Wilcoxon-Mann-Whitney rank sum tests were utilized for bivariate analysis, and multiple logistic regression for multivariate analysis.

**Results:**

From January 2014 to December 2014, there were 185 cases of perinatal death, of which 36% (*n* = 69) were macerated stillbirths, 40% (*n* = 76) were fresh stillbirths, and 25% (*n* = 47) were neonatal deaths. The rate of perinatal death among all deliveries at the institution was 35.5 per 1000 deliveries. Factors associated with increased odds perinatal death included: prematurity (adjusted odds ratio (aOR) 19.7, 95% confidence interval (CI) 7.2–49.2), breech presentation (aOR 7.0, CI 1.4–35.5), multiple gestation (aOR 4.0, CI 1.1–13.9), cesarean delivery (aOR 3.8, CI 2.3–6.4) and low birth weight (aOR 2.5, CI 1.1–5.3). Analysis by subtype of perinatal death revealed distinct associations with the aforementioned risk factors, in particular for antepartum hemorrhage, which was only associated with fresh stillbirths (aOR 6.7, CI 1.6–28.8), and low birth weight.

**Conclusions:**

The rate of perinatal death at our rural hospital site was higher than national targets, and these deaths were associated with prematurity, low birth weight, breech presentation, multiple gestation, and cesarean delivery. This data and the approach utilized to acquire it can be leveraged to inform targeted interventions to reduce the rate of stillbirths and neonatal deaths in similar low resource settings.

## Introduction

Perinatal deaths, defined as a composite of stillbirths and neonatal deaths, are unequally distributed globally as evidenced by upwards of 98% occurring in low- and middle-income countries [1, 2]. Of the estimated 2.7 million neonatal deaths and 2.6 million stillbirths that occur annually, the majority are likely avertable [3]. The Every Newborn Action Plan, which was launched in 2014 by the World Health Organization and United Nations International Children’s Emergency Fund, strives to promote progress in preventing such deaths across the globe and specifically to achieve 10 or fewer stillbirths among 1000 total births and 10 or fewer newborn deaths among 1000 live births by 2035 [4]. However, significant work remains to be done to close the gap between current circumstances and what is desired.

Uganda is among the top fifty countries with the highest burdens of perinatal deaths [5, 6]. In 2015 its stillbirth rate was 21 per 1000 births and neonatal death rate was 19 per 1000 live births, approximately double of the targets set forward in the Every Newborn Action Plan [5, 6]. While important advancements are happening on the national level such as the development of a strategic plan, regional and facility-level efforts vary secondary to numerous factors ranging from access to specialists to antenatal care coverage [7, 8]. An estimated 52% of deliveries in rural Uganda occur in hospital settings as compared to close to 90% in urban areas [7]. Local differences could theoretically result in distinct case mixes between rural and urban care settings, which are crucial to understand given that over three quarters of Uganda’s population lives in rural locations [9].

In light of the limited data on perinatal deaths from rural hospitals, the goal of our study was to identify risk factors for perinatal death in one of Uganda’s rurally located district hospitals using routinely collected clinical data.

## Methods

Tororo District Hospital is a 200-bed government-owned facility located in eastern Uganda. According to the 2014 census, the population of Tororo was approximately 517,000 with 86% living in a rural households [8]. The hospital serves a catchment area of over 500,000 people extending to the Kenya-Uganda border and beyond. Supervised by the one to two physicians covering the hospital inpatient and outpatient service, two to three birth attendants staff the six-bed labor suite. One operating theatre with two rooms serves all surgical needs. Providers utilize fetoscopes for intermittent fetal heart rate, partographs to monitor labor progress, and limited equipment (e.g. ambubags) for neonatal resuscitation. The skilled birth attendants complete handwritten birth registers on admission and discharge to collect maternal and neonatal data. Gestational age was primarily ascertained using patient reported dating and use of a pregnancy wheel. Birth attendants categorized demised fetuses prior to 24 weeks gestation as miscarriages, and at or beyond 24 weeks as stillbirths. Neonatal deaths within the first 28 days of life that occurred in the hospital and those of discharged neonates who were confirmed to be dead in that same time frame are counted as perinatal deaths per hospital protocol.

A retrospective case-control study was performed with anonymous data from all available birth register data for January 2014 to December 2014. This data had been collected and digitized by author ND for quality improvement purposes. Cases were maternal admissions that resulted in perinatal death as defined as stillbirth or neonatal death as documented in the birth registry. Of note, macerated stillbirths were those with findings suggestive of death greater than 8–24 h prior to delivery (e.g. skin desquamation) whereas fresh stillbirths were those without such findings [10, 11]. Control patients, collected in a 2:1 ratio, were the admissions immediately prior and immediately after the perinatal death case that had liveborn neonates who survived to discharge and was not discovered to have died within 28 days of delivery. We utilized this approach to account for temporal factors such as hospital census, staffing ratios, and medication availability which can be more variable in resource-limited settings. As no additional data was available outside of the birth registers for clarification, illegible data were excluded from the database.

Data on maternal factors including maternal age, parity, prior cesarean and human immunodeficiency virus (HIV) status were collected. Parity of more than 3 births prior to the index pregnancy was evaluated to assess association with grand parity. Pregnancy level data collected included multiple gestation, preeclampsia, antepartum hemorrhage, infection, breech presentation, cord prolapse, mode of delivery, and preterm gestation. Birth weights were collected, and low birth weight was defined as less than 2.5 kg. Data was only excluded if it was illegible.

This study was approved by the Tororo General Hospital Ethics Committee and the Medical Superintendent. The study was considered exempt from the University of California San Francisco Institutional Review Board review as the data was de-identified. Descriptive statistics were done using StataSE 15 (StataCorp, College Station, TX). Continuous variables were analyzed using t-tests if parametric and Wilcoxon-Mann-Whitney rank sum tests if non-parametric. Categorical variables were analyzed using Chi square and Fisher’s exact tests as was indicated by cell frequencies. Multiple logistic regression was used to each variables’ association with the outcome of either stillbirth or neonatal death as well as with each subtype of perinatal death. The models were controlled for the nonmodifiable predictor variables of maternal age, nulliparity, twin pregnancy, and prematurity. Collinearity was assessed for all predictor variables prior to application of these models to confirm that variance inflation factors were less than ten. We employed an available case analysis to missing data and thus include relevant denominators for variables with missing data. A *p* value of < 0.05 was considered statistically significant.

No funding was received for the realization of this study. We utilized Strengthening the Reporting of Observational Studies in Epidemiology (STROBE) guidelines for case-control studies for preparation of this manuscript.

## Results

**Fig. 1:**
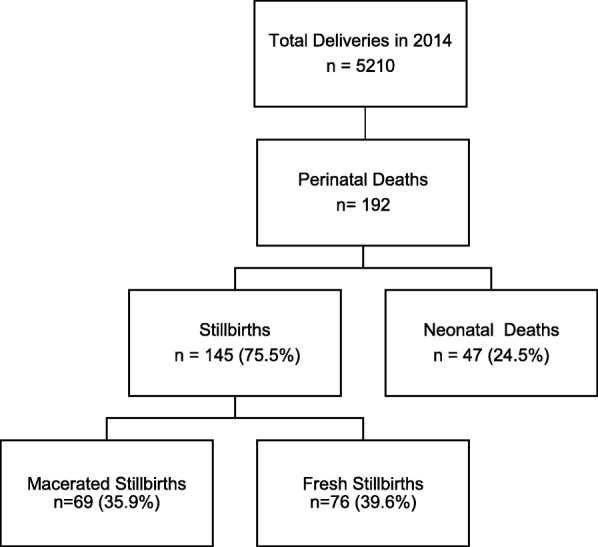
Type of perinatal deaths among case pregnancies on the level of neonate (percentage of total number of perinatal deaths)

Tororo General Hospital had approximately 5210 deliveries from January 2014 to December 2014. There were 46 weeks of birth register data available for our study and approximately 90% was legible. Among these registers, there were 185 case pregnancies with perinatal death and 354 control pregnancies. 24 of the included pregnancies were of twins, 19 of which were cases with loss of both twins in 7 (36.8%) and 12 with loss of one twin (63.2%). The distribution of perinatal deaths by type (i.e. macerated stillbirth, fresh stillbirth, or neonatal death) is shown in Fig. 1. The calculated stillbirth rate was 26.3 per 1000 total deliveries, and the neonatal death rate of 8.9 per 1000 live deliveries.

Comparison between case and control pregnancies of the variables analyzed is shown in Table 1. The mean age for women who experienced a perinatal loss was 1.4 years older than those who did not (*p* = 0.02). The following factors were associated with increased odds of perinatal death: prematurity, low birth weight, multiple gestation, breech presentation, antepartum hemorrhage, cesarean delivery and cord prolapse. Factors associated with decreased odds of perinatal death included having more than 3 prior births and presenting in spontaneous labor. There were no significant associations between perinatal death and nulliparity, HIV infection, preeclampsia, or prior cesarean. Multiple logistic regression demonstrated that the adjusted OR for all of the preceding significant associations remained statistically significant with the sole exception of antepartum hemorrhage.

**Table 1:** Maternal, pregnancy, fetal and neonatal characteristics of case and control pregnancies with statistically significant associations (*p* < 0.05) bolded

	Control pregnancies *N* = 354^a^	Case pregnancies *N* = 185^a^	
	n (%) or Median (IQR)	n (%) or Median (IQR)	Adjusted OR (CI)^b^
Maternal Age (years)	23 (19—29)	25 (20–30)	–
Nulliparity	132/344 (38.4%)	62/179 (34.6%)	1.1 (0.7–1.1)
More than 3 prior births	**277/344 (80.5%)**	**116/179 (64.8%)**	**0.3 (0.2–0.7)**
Prior cesarean	8/348 (2.3%)	4 (2.2%)	1.3 (0.4–4.7)
HIV	26/342 (7.6%)	9/176 (5.1%)	0.5 (0.2–1.2)
Twin pregnancy	**5 (1.4%)**	**19 (10.3%)**	**4.0 (1.1–13.9)**
Preeclampsia	2/343 (0.6%)	0/179 (0%)	–
Antepartum hemorrhage	**4/343 (1.2%)**	**8/179 (4.5%)**	3.2 (0.9–12.2)
Infection	7/343 (2.0%)	7/179 (3.9%)	2.5 (0.8–7.7)
Breech	**2/348 (0.6%)**	**8 (4.3%)**	**7.0** **(1.4–35.5)**
Cord prolapse	**0/343 (0%)**	**7/179 (3.9%)**	–
Normal labor	**317/343 (92.4%)**	**97/179 (54.2%)**	**0.1 (0.1—0.2)**
Cesarean delivery	**35/347 (10.0%)**	**46/182 (25.3%)**	**3.8 (2.3–6.4)**
Prematurity	**5/348 (1.4%)**	**40/179 (22.2%)**	**18.9** **(7.2–49.2)**
Birth weight (kilograms, kg)^c^	**3.1 (2.7—3.3)**	**2.8 (1.7–3.2)**	**–**
Low birth weight (< 2.5 kg)^c^	**21/338 (3.7%)**	**56 (37.3%)**	**2.5 (1.1–5.3)**

*OR* Odds ratio, *CI* Confidence interval, *IQR* Interquartile Range

^a^Denominators noted in cells when distinct from control and case N secondary to missing data

^b^Adjusted for maternal age in years, nulliparity, twin pregnancy, and prematurity

^c^Data was analyzed on the level of the fetus/neonate rather than pregnancy

To assess for temporal association of factors with perinatal death (i.e. preceding, during or after labor), macerated stillbirths, fresh stillbirths and neonatal deaths were each compared to control pregnancies as shown in Table 2. Only singleton pregnancies were included in this subgroup analysis given that some twin dyads had different types of perinatal death (e.g. twin A was a stillbirth; twin B was a neonatal demise). Breech presentation, prematurity, low birth weight and cord prolapse were significantly associated with increased odds of macerated stillbirths in contrast to the other categorical variables. All of these as well as antepartum hemorrhage and cesarean section were associated with increased odds of fresh stillbirth. Only prematurity, breech presentation, cesarean delivery, and cord prolapse were associated with increased odds of neonatal death with prematurity having the strongest association with neonatal death (aOR 36.2 compared to 18.1 for macerated stillbirth and 7.5 for fresh stillbirth).

**Table 2:** Adjusted odds ratios for subtypes of perinatal death by maternal, pregnancy, fetal and neonatal characteristics as compared to controls among all singleton pregnancies with statistically significant associations (*p* < 0.05) bolded

	Macerated Stillbirths *N* = 64	Fresh Stillbirths *N* = 61	Neonatal Deaths *N* = 41
	Adjusted OR (CI) ^a^	Adjusted OR (CI) ^a^	Adjusted OR (CI) ^a^
More than 3 prior births	**0.2 (0.1–0.5)**	**0.4 (0.1–0.9)**	0.6 (0.2–2.0)
Antepartum hemorrhage	1.7 (0.2–12.5)	**6.7 (1.6–28.8)**	1.0 (0.1–19.9)
Breech	**18.4 (6.3–54.1)**	**21.4 (2.2–204.5)**	**30.1 (2.5–373.2)**
Cord prolapse	**1.0**^**b**^	**1.0**^**b**^	**1.0**^**b**^
Normal labor	**0.1 (0.0–0.2)**	**0.1 (0.1–0.22)**	0.5 (0.2–1.4)
Cesarean delivery	0.8 (0.3–2.4)	**6.5 (3.4–12.6)**	**2.5 (2.5–13.3)**
Prematurity	**18.1 (6.2–53.1)**	**7.5 (2.2–25.9)**	**36.2 (11.9–110.2)**
Low birth weight (< 2.5 kg)^c^	**6.8 (2.6–17.9)**	**1.3 (0.4–4.9)**	1.6 (0.3–7.4)

*OR*, odds ratio, *CI*, confidence interval

^a^Adjusted for maternal age in years, nulliparity, and prematurity

^b^Predicts perinatal death subtype perfectly

^c^Data was analyzed on the level of the fetus/neonate rather than pregnancy

## Discussion

In summary, we investigated risk factors for perinatal death in a rural hospital in Uganda, a common care setting from where there is limited patient-level data. We found a stillbirth rate of 26.3 per 1000 total deliveries, which was over 25% higher than the WHO-estimated national average of 21.0 at the time and greater than 2.5-fold higher than the Every Newborn Action Plan target of 10 [5, 6]. Prematurity (aOR 19.7), breech presentation (aOR 7.0), and twin gestation (aOR 4.0) had the strongest associations with perinatal death. We did find that some risk factors to be associated with certain subtypes of perinatal death, specifically fresh stillbirths with antepartum hemorrhage and neonatal deaths being more strongly associated with prematurity than either type of stillbirth. Given the limited resources at the study site and other facilities in rural settings, these findings provide important information that can guide further inquiry and initiatives that could improve perinatal outcomes.

Our findings complement the limited data in literature on perinatal mortality in rural Uganda. Data from a cross sectional study conducted in rural Eastern Uganda in 2013 with women who delivered within the year prior found increased risk of neonatal death with grand multiparity, increasing maternal age and low birth weight, but women who experienced a stillbirth were notably excluded and limited obstetric data was assessed aside from number of antenatal visits and place of delivery [12]. Our findings are notably distinct from studies in more urban settings in Uganda. In a prospective cohort study in 2013–2014 of referral hospitals in the urban capital Kampala and the smaller semi-urban town of Jinja, obstructed labor, uterine rupture, antepartum hemorrhage and hypertensive disorders of pregnancy were noted to be the most frequent diagnoses associated with perinatal deaths [13]. Our findings also differ from a retrospective study from a rural hospital in southwestern Uganda that took place from 2009 to 2011 that did find similar associations of perinatal death with prematurity and birth weight but also with maternal HIV positive status, which we did not see in our study, which could be secondary to a higher prevalence in that cohort than ours [14]. Future prospective studies directly comparing different practice settings are needed to further investigate these differences.

Strengths of our study include the detailed review of the birth registry, a readily available and routinely updated resource whose maintenance preceded and continues beyond the study period. The number of stillbirths ascertained in the study database exceeded the number in the hospital annual report, which only noted 65 fresh stillbirths, 66 macerated stillbirths and 35 neonatal deaths in 2014. This finding suggests that the majority of perinatal births had been captured perhaps more successfully than other tracking systems used. Use of temporally related controls was another strength of the study to help ensure that cases and controls had similar circumstantial factors that are known to impact perinatal outcomes. The study population’s extrapolated annual stillbirth rate, similar to hospitals across the country in a 2014 report, was higher than national rate, which supports the utility of focusing on in-facility perinatal deaths [8]. Another strength is our analysis on differing association of risk factors for the subtype of perinatal death, that is macerated stillbirth, fresh stillbirth, and neonatal death, which could elucidate potential opportunities for intervention to prevent stillbirths and neonatal deaths after presentation to the hospital.

An important limitation of our study was the quantity and quality of data that was available in the birth registers. Given the small sample size, the confidence intervals of certain variables were wide and thus need to be interpreted with caution. While an assumption of the study was that all the fields were being accurately completed, there is the possibility that factors were variably reported due to the numerous other responsibilities held by those completing the registers and the reality that important factors such as congenital anomalies were not captured at all. Ability to interpret the handwriting on the registers limited the ability to include data. Also, neonatal deaths that occurred after discharge but before 4 weeks of life were only captured if the deceased newborn was brought back to the hospital, thus resulting in likely under reporting of this outcome. Additionally, the lack of data on fetal heart rate at the time of admission limited the ability to stratify stillbirths that occurred during engagement in care and those that occurred prior, which would afford a greater appreciation for the impact inpatient care could have had. These limitations highlight opportunities to advocate for strengthening the existing birth registry system (e.g. independent data abstractors, quality assurance mechanisms, postnatal follow up), a common issue in low-resource settings.

## Conclusions

Our study in a district hospital in rural Uganda identified statistically significant associations of perinatal death with the following risk factors: multiple gestation, low birth weight, prematurity, breech presentation, cesarean delivery. This data and the approach can be utilized to acquire it can be leveraged to inform targeted interventions to reduce the rate of stillbirths and neonatal deaths at the study site and beyond.

In their article on trends and risk factors of perinatal deaths in Eastern Uganda, Kujala et al. write, “The slow decline in mortality rates and easily identifiable risk factors calls for improving quality of care at birth and a rethinking of how to address obstetric risks, potentially a revival of the risk approach of antenatal care” [15]. Our study used routinely collected clinical data to identify important associations with perinatal deaths, which is an approach that can be used to guide health systems strengthening interventions to optimize the care for patients with relevant factors. For example, targeted audits of the perinatal deaths have been demonstrated to decrease such losses in a peri-urban setting in Kampala, Uganda, but the authors acknowledged that only approximately one-third of cases were reviewed because of “lack of time or lack of clinical case notes” [16]. Using data such as those presented in our study could help focus the attention of such audits given the limited resources available to execute them. For example, an audit focused on fetal number, heartbeat and presentation on admission as well as inpatient management of breech presentation, multiple gestation and patients in need of cesarean section could allow for local providers to more efficiently identify potential opportunities for improving outcomes for such cases.

In summary, we report that the rate of perinatal death in a non-academic rural hospital setting is higher than that based on regional reports and identified associated risk factors for adverse outcome. These findings can be used to help prioritize interventions to improve perinatal outcomes in similar, low-resource settings.

## Data Availability

The datasets used and analyzed during the current study are available from the corresponding author on reasonable request.

## Abbreviations

aOR: Adjusted odds ratio
CI: Confidence interval
HIV: Human immunodeficiency virus
OR: Odds ratio
